# Hearty Suppers

**Published:** 1871-02

**Authors:** 


### HEARTY SUPPERS.

It requires about five hours for the stomach to work on an ordinary meal and pass it out of itself, when it falls into a state of repose. Hence, if a man eats three times a day, his stomach must work fifteen hours out of the twenty-four. But the multitude of mechanics who are wildly clamorous for only "eight hours a day," are the very ones who, while they are angered at being required by others to work more than eight hours a day, do not hesitate to impose on their stomachs fifteen hours' work; nearly double. After a night's sleep, we wake up with a certain amount of bodily vigor, which is faithfully portioned out to every muscle of the system, and every set of muscles, each its rightful share; the stomach among others. When the external body gets weary after a long day's work, the stomach bears its share of the fatigue; but if when the body is weary with the day's toil, we put it to bed, giving the stomach meanwhile a five hours' task, which must be performed, we impose upon the very best friend we have, the one that gives us one of the largest amounts of earthly enjoyments; and if this overtaxing is continued, it must as certainly wear out prematurely as the body itself will, if it is overworked every day. And if persons eat between meals, then the stomach has no rest from breakfast in the morning, until one, two, three, or four o'clock next day; hence it is that so many persons have dyspepsia, the stomach is worked so much and so constantly, that it becomes too weak to work at all. It is to be hoped that every intelligent parent will press these things on the attention of their children as a matter of conscience, because dyspepsia, like consumption, has its foundations laid in a large majority of cases, during the " teens " of life.

